# Molecular characterization of *Plasmodium falciparum* DNA-3-methyladenine glycosylase

**DOI:** 10.1186/s12936-020-03355-w

**Published:** 2020-08-06

**Authors:** Nattapon Pinthong, Paviga Limudomporn, Jitlada Vasuvat, Poom Adisakwattana, Pongruj Rattaprasert, Porntip Chavalitshewinkoon-Petmitr

**Affiliations:** 1 Department of Protozoology, Faculty of Tropical Medicine, Mahidol University, Bangkok, Thailand; 2 Department of Zoology, Faculty of Science, Kasetsart University, Bangkok, Thailand; 3 Department of Helminthology, Faculty of Tropical Medicine, Mahidol University, Bangkok, Thailand

**Keywords:** *Plasmodium falciparum*, DNA-3-methyladenine glycosylase, DNA repair, Malaria

## Abstract

**Background:**

The emergence of artemisinin-resistant malaria parasites highlights the need for novel drugs and their targets. Alkylation of purine bases can hinder DNA replication and if unresolved would eventually result in cell death. DNA-3-methyladenine glycosylase (MAG) is responsible for the repair of those alkylated bases. *Plasmodium falciparum* (*Pf*) MAG was characterized for its potential for development as an anti-malarial candidate.

**Methods:**

Native *Pf*MAG from crude extract of chloroquine- and pyrimethamine-resistant *P*. *falciparum* K1 strain was partially purified using three chromatographic procedures. From bio-informatics analysis, primers were designed for amplification, insertion into pBAD202/D-TOPO and heterologous expression in *Escherichia coli* of recombinant *Pf*MAG. Functional and biochemical properties of the recombinant enzyme were characterized.

**Results:**

*Pf*MAG activity was most prominent in parasite schizont stages, with a specific activity of 147 U/mg (partially purified) protein. K1 *PfMAG* contained an insertion of AAT (coding for asparagine) compared to 3D7 strain and 16% similarity to the human enzyme. Recombinant *Pf*MAG (74 kDa) was twice as large as the human enzyme, preferred double-stranded DNA substrate, and demonstrated glycosylase activity over a pH range of 4–9, optimal salt concentration of 100–200 mM NaCl but reduced activity at 250 mM NaCl, no requirement for divalent cations, which were inhibitory in a dose-dependent manner.

**Conclusion:**

*Pf*MAG activity increased with parasite development being highest in the schizont stages. K1 *PfMAG* contained an indel AAT (asparagine) not present in 3D7 strain and the recombinant enzyme was twice as large as the human enzyme. Recombinant *Pf*MAG had a wide range of optimal pH activity, and was inhibited at high (250 mM) NaCl concentration as well as by divalent cations. The properties of *Pf*MAG provide basic data that should be of assistance in developing anti-malarials against this potential parasite target.

## Background

Malaria is one of the major infectious diseases threatening two-thirds of the world’s population, especially those living in tropical and sub-tropical regions, imposing both a disease and economic burden in these countries [1]. The World Health Organization (WHO) reported 228 million new cases of malaria in 2018, with 97% of the infection in sub-Saharan Africa caused by *Plasmodium falciparum* and resulting in 405,000 deaths, mainly of children [2].

*Plasmodium falciparum* causes most severity in terms of clinical pathology and complication in treatment as it readily develops resistance to all existing anti-malarial agents, including most recently the artemisinins [3, 4], highlighting the urgent need for identification of new parasite targets and development of safe and effective novel drugs targeting them. Although a malaria vaccine has recently become available, it only provides partial protection [5], and chemotherapeutic agents still play an essential role in malaria treatment and prevention.

Among the various parasite targets being studied for drug development, enzymes in *P. falciparum* DNA repair pathway present potential drugable targets, including *P. falciparum* uracil DNA glycosylase (*Pf*UGD) [6], *P. falciparum* DNA polymerase delta (*Pf*Polδ) [7] and *P. falciparum* ATP-dependent DNA helicase RuvB3 (*Pf*RuvB3) [8]. The parasite genome lacks genes encoding DNA repair enzymes in the non-homologous end joining pathway, but previous identification of *Pf*Polδ [7] suggests parasite base excision repair mechanism might rely mainly on a long patch repair pathway [9].

The high A–T content of the malaria parasite genome implies the potential of these regions being modified (alkylated), thereby the need of a parasite repair enzyme. DNA-3-methyladenine DNA glycosylase (MAG), a single sub-unit monofunctional DNA repair enzyme, belongs to an alkyladenine DNA glycosylase (AAG) superfamily, characterized by an antiparallel β-sheet and flanked by α-helices [10]. The enzyme is capable of removing 3-methyladenine (m^3^A) as well as other cyclic adducts in DNA, such as 1,*N*^6^ethenoadenine (εA), 3,*N*^*4*^-ethenocytosine (εC), *N*^*2*^,3-ethenoguaine (*N*^*2*^,3-εG), and l,*N*^*2*^-ethenoguanine (1,*N*^*2*^-εG) [11]. MAG orthologues are present in *Escherichia coli*, *Saccharomyces cerevisiae*, rodents, humans, and plants [12, 13]. It is also known as *N*-methylpurine DNA glycosylase (MPG) due to its versatility in accommodating a variety of substrates in the active site [14]. MAG knockdown in animal models and cell cultures results in a modulation of sensitivity to alkylating agents [15, 16]. In addition, 3-methyladenine and 1,*N*^*6*^-ethenoadenine are able to inhibit progression of DNA replication fork and thereby the DNA replication process [17–19]. In *P. falciparum*, after decades of debate [20–22] the existence of methylated cytosines (me^5^C) were finally identified in genomic DNA by the use of unbiased bisulfite conversion coupled with deep sequencing [23]. Recently, a hydroxymethylcytosine-like modification was identified at a higher extent compared with me^5^C and was linked to *P. falciparum* gene expression [24]. On the other hand, there is no available information to date with regards to purine methylation of the parasite. However, a gene encoding *Pf*MAG was found located on chromosome 14 of chloroquine- and pyrimethamine-sensitive *P. falciparum* 3D7 strain comprising of 1506 nucleotides coding 501 amino acids (PlasmoDB: PF3D7_1467100).

Since MAG plays an important role in DNA repair and little is known regarding *Pf*MAG, this provides an opportunity to study its properties as a potential target for anti-malarial drug development [25]. Here, native *Pf*MAG was partially purified from parasite crude extract to verify its expression in asexual parasites, and recombinant *Pf*MAG was heterologously produced to allow further characterization of biochemical and functional properties.

## Methods

### *Pf*MAG activity determination of *Plasmodium falciparum* asexual stages

*Plasmodium falciparum* K1 strain, a chloroquine- and pyrimethamine-resistant strain isolated in Thailand [26], was cultivated in RPMI 1640 medium (Invitrogen™, CA, USA) supplemented with 10% human serum and human red blood cells (RBCs) at 37 °C using the candle jar method [27]. Media was changed daily and morphology and parasitaemia was observed under a light microscope (1,000× magnification) using Giemsa-stained thin blood film. Parasite culture was initiated with 2% parasitaemia of ring forms obtained from sorbitol synchronization [28]. Ring, trophozoite and schizont stages were separately harvested when parasitaemia reached 20–30%. Each parasite stage was prepared by incubating sedimented, infected RBCs with an equal volume of phosphate-buffered saline pH 7.6 (PBS) containing 0.15% (w/v) saponin at 37 °C for 20 min. Cell suspension was washed twice with PBS by centrifugation at 700×*g* at 25 °C for 10 min and parasite pellet was stored at − 80 °C until used.

Approximately 0.5 ml aliquot of each stage of parasite pellet was resuspended in 4 volumes of extraction buffer (50 mM Tris–HCl pH 7.6 containing 1 mM EDTA, 2 mM DTT, 0.01% NP40 and 1 mM PMSF) and cells were fragmented in a Dounce homogenizer. An equal volume of dilution buffer (50 mM Tris–HCl pH 7.6 containing 1 mM EDTA, 2 mM DTT, 20% (w/v) sucrose, 0.01% NP40 and 1 mM PMSF) was added to the sample and 3 M KCl was slowly added to the mixture to a final concentration of 0.5 M KCl while stirring on ice for 30 min. Then the sample was centrifuged at 100,000×*g* at 4°C for 45 min, supernatant dialysed at 4 °C overnight against buffer A (25 mM Tris–HCl pH 8.5 containing 1 mM EDTA, 1 mM PMSF, 1 mM DTT, 5% sucrose, 20% glycerol, and 0.01% NP40) and used for assay of *Pf*MAG activity.

### Partial purification of native *Pf*MAG

Parasite culture for partial purification of native *Pf*MAG was carried out using a large-scale culture method [29]. *Plasmodium falciparum* cultures, containing mostly trophozoite and schizont stages, were harvested at > 20% parasitaemia by centrifugation at 500×*g* for 10 min at 25 °C. Parasite pellet (2 ml) was resuspended in extraction buffer, homogenized and parasite extract prepared as described above.

Parasite extract was loaded onto a HiTrap Q column (GE Healthcare, USA) equilibrated with buffer A and column then was washed with 10 ml of buffer A and proteins were eluted using 10 ml of a 0–1 M KCl linear gradient in buffer A. Fractions of 250 μl were collected and 5 ml aliquot of each fraction was tested for glycosylase activity. Fractions containing *Pf*MAG activity were pooled and dialyzed against buffer B (50 mM Tris pH 8.0 containing 1 mM PMSF, 2 mM DTT, 1 mM EDTA, 5% sucrose, 20% glycerol, and 0.01% NP40) overnight at 4 °C and then loaded onto HiTrap Capto S column (GE Healthcare) equilibrated with buffer B. The column was washed with buffer B and proteins were eluted with 15 ml of a 0–1 M KCl linear gradient in buffer B. Fractions of 250 μl were collected, assayed for *Pf*MAG activity and pooled fractions dialyzed against buffer B, then loaded onto Hitrap Heparin column (GE Healthcare) equilibrated with buffer B. Column was washed with buffer B and proteins eluted with 10 ml of a 0–1 M KCl linear gradient in buffer B. Fractions containing *Pf*MAG activity were pooled and termed native *Pf*MAG.

### *Pf*MAG glycosylase assay

Fluorescent-labelled 27-mer oligonucleotide 5ʹ-[6FAM] CGATTAGCATCCTXCCTTCGTCGTCTCCAT-3ʹ (where X = εA) (Gene Link™; NY, USA) was annealed to its complementary strand 5ʹ-ATGGAGACGACGAAG GTAGGATGCTAATCG-3ʹ at 1:2 molar ratios in 100 μl reaction containing TE buffer (10 mM Tris–HCl pH 8 containing 1 mM EDTA,). The annealing process was carried out by heating at 95 °C for 5 min and cooling to ambient temperature over a period of 30 min, then the annealed substrate was stored at 4 °C until used.

*Pf*MAG activity assay mixture (25 μl) containing 50 mM sodium phosphate pH 7, 1 mM EDTA, 1 mM DTT, 100 mM NaCl, 200 μg/ml BSA, 0.5 μM oligoduplex substrate and 1.5 mM of recombinant *Pf*MAG was incubated at 37 °C for 30 min, then reaction terminated by 200 mM NaOH and heating at 95 °C for 5 min. The solution was mixed with an equal volume of loading buffer (98% formamide, 10 mM EDTA and xylene cyanol FF and bromophenol blue dyes) and resolved on 16% urea PAGE. The 27-mer and 13-mer products were visualized by VersaDoc 4000 MP (Biorad, USA) and quantified using an image-analysis software (Image Lab; Biorad, USA). Recombinant *Pf*MAG activity was evaluated for substrate preference and optimal incubation time, pH, and salt and divalent cations concentrations. One U MAG activity is defined as the amount of *Pf*MAG required to release 1 pmol of 1,*N*^6^ethenoadenine in 1 min at 37 °C.

In addition to double-stranded DNA as substrate, fluorescent-labelled, single-stranded 27-mer oligonucleotide was tested as DNA substrate of recombinant *Pf*MAG compared to human DNA-3-methyladenine glycosylase (hAAG) (specificity of 5000 U/mg; Sigma-Aldrich, Germany). Enzyme incubation time varied from 10 to 90 min. Reactions were carried out as described above. *Pf*MAG activity was tested in presence of 50 mM citrate-sodium citrate buffer pH 3–5, phosphate-citrate buffer pH 6–7 and Tris–HCl pH 8–9. *Pf*MAG activity was determined in 50–500 mM NaCl under standard assay reaction described above, as well in the presence of Fe^2+^, Mg^2+^ and Zn^2+^ (0–3 mM) in the absence of EDTA. Relative activity of *Pf*MAG was determined as amount of product divided by total amounts of substrate and product.

### Expression analysis of *PfMAG* using SYBR Green quantitative (q)PCR

Total RNA was isolated from ring, growing trophozoite and schizont stages of *P. falciparum* using an Easy-Spin™ (DNA-free) and total RNA extraction kit (iNtRON Biotechnology, South Korea). Purity of RNA in eluted samples (50 µl) of was assessed using a NanoDrop™ spectrophotometer (Thermo Scientific, USA). Reverse transcription was carried out using a Maxime™ RT PreMix (Oligo (dT)15 Primer) kit (iNtRON Biotechnology, South Korea) in a reaction volume of 20 µl at 45 °C for 60 min. PCR primers were designed based on alignment of a *P*. *falciparum* fragment (NCBI Accession No. XM_001348777) (Table 1), with serine-tRNA ligase as an internal control gene [30]. All amplifications were performed using a LightCycler^®^ FastStart DNA Master SYBR Green I (Roche Applied Science, Germany). The following thermocycling conditions were used: 10 min at 95 °C for initial denaturation and enzyme activation; 45 cycles of 95 °C for 10 s, 55 °C for 5 s and 72 °C for 10 s; followed by melting curve analysis of 65–95 °C. Melting temperature of *PfMAG* and *serine*-*tRNA ligase* cDNA was 72.9 and 74.5 °C, respectively. Relative quantification of *PfMAG* expression employed a 2^−∆∆Ct^ method [31]. Three independent experiments were performed in duplicate for each parasite sample.

**Table 1:** Primers used in expression analysis of *PfMAG* using SYBR Green quantitative PCR

Primer name	Sequence	Reference
PfMAGexpressF	GGAACCAACAAGGGAACATATCA	In house
PfMAGexpressR	TTGTTACACATCCAGGACCACT	In house
s-tRNA syn F	AAGTAGCAGGTCATCGTGGTT	[30]
s-tRNA syn R	TTCGGCACATTCTTCCATAA	[30]

### PCR amplification of *PfMAG*

DNA of *P. falciparum* strain K1 was extracted using QIAamp DNA Blood Mini Kit (Qiagen, USA). *PfMAG* was amplified using forward primer 5ʹ-CACCATGGAA AAAATGAACGAATTC-3ʹ designed from the start codon and incorporating a specific sequence at 5ʹ end (CACC) for unidirectional cloning and reverse primer 5ʹ-TTTGGG AAAAATAGATACGGATGG-3ʹ designed for full-length gene amplification; primers design used *P*. *falciparum* strain 3D7 as template (NCBI accession no. XM_001348777). Amplification was performed (using Phusion™ High—Fidelity PCR Kit; Finzyme OY, Finland) as follows: 98 °C for 5 min; 35 cycles of 98 °C for 30 s, 55 °C for 30 s and 72 °C for 30 s; with a final heating at 72 °C for 3 min. 

### Cloning, heterologous expression and purification of recombinant *Pf*MAG

Amplified full-length *PfMAG* was ligated into expression vector pBAD202/D-TOPO (Invitrogen, USA). The constructed recombinant plasmid was verified by DNA sequencing and compared to its known *P. falciparum* 3D7 counterpart. *Escherichia coli* LMG 194 harbouring recombinant pBAD-PfMAG was grown in LB media containing 50 µg/ml kanamycin sulfate at 37 °C, with shaking at 200 rpm for 2 h. When A_600__nm_ of the culture reached 0.4, incubation temperature was reduced to 15 °C and shaking was continued for a further 1 h. L-arabinose (0.02% w/v) was then added to induce recombinant protein expression and the culture was shaken for 16 h. Following sedimentation, bacterial pellet was suspended in lysis buffer (50 mM NaH_2_PO_4_ pH 8.0 containing 300 mM NaCl and 10 mM imidazole) and lysed by sonication on ice for 10 min. Then the sample was centrifuged at 10,000×*g* at 4 °C for 30 min, supernatant added to an equal volume of lysis buffer and loaded onto a HisTrap HP column (GE Healthcare) pre-equilibrated with lysis buffer. The column was washed with washing buffer (50 mM NaH_2_PO_4_ pH 8.0 containing 300 mM NaCl and 50 mM imidazole) and protein eluted with a linear 10–500 mM imidazole gradient. Fractions (250 μl) with enzyme activity were pooled and subjected to 12% SDS-PAGE, staining with Coomassie blue R250 and western blotting. The latter employed mouse anti-His primary antibody (Invitrogen, USA) (1:3000 dilution) and rabbit HRP-conjugated anti-mouse IgG antibody (1:5000 dilution). Immunoreactive protein band was visualized by treatment with a mixture of hydrogen peroxide and 3,3′-diaminobenzidine tetrahydrochloride (DAB).

## 3D structure prediction of *Pf*MAG

In view of a lack of 3D structural models of *Pf*MAG, a predicted structure was constructed using the 502-amino acid sequence of PfMAG K1 as input for simulation of *Pf*MAG by a protein threading method using I-TASSER server [32–34] and a Pymol software to align *Pf*MAG and superimpose on human AAG structure.

## Results

### Partial purification of native *Pf*MAG

**Fig. 1:**
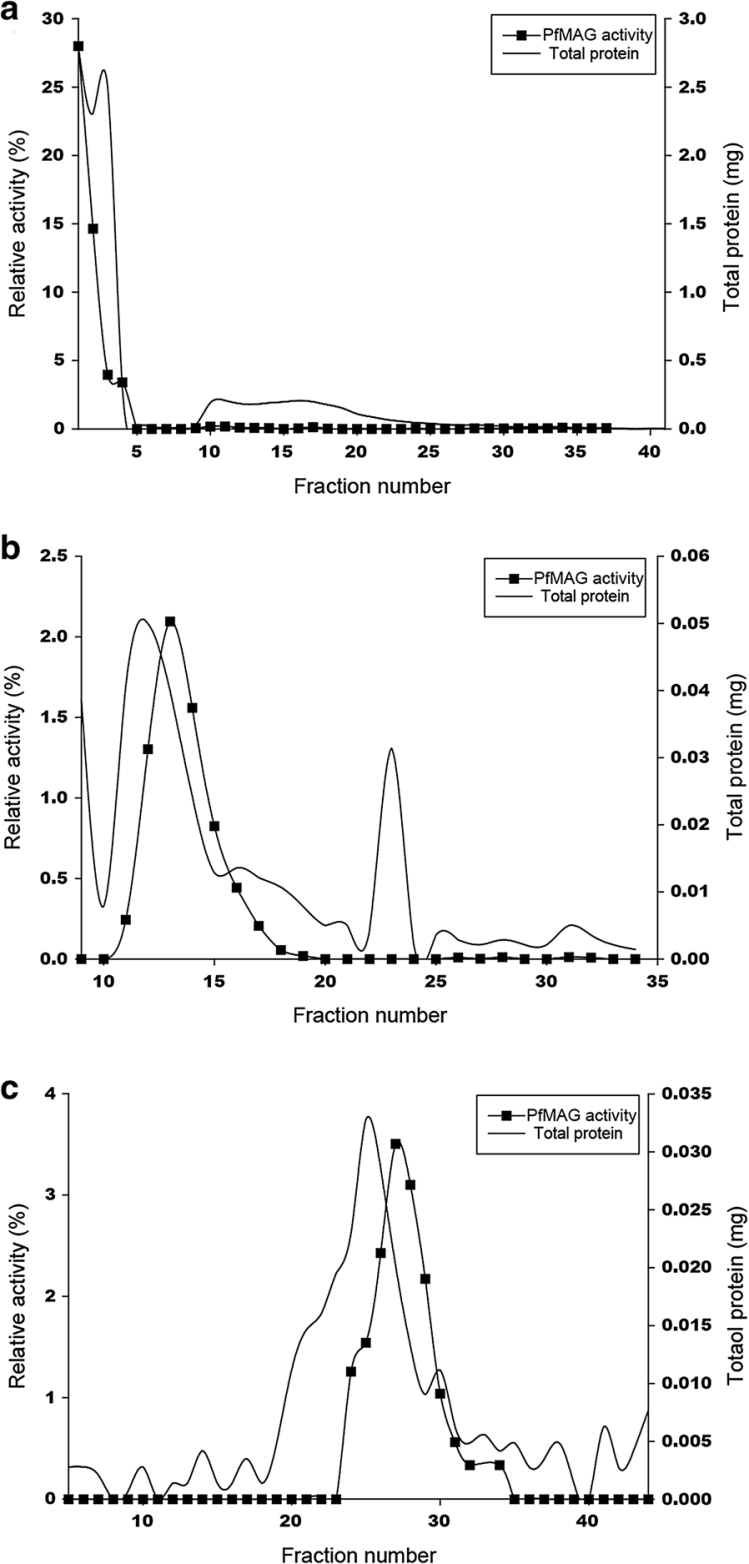
Partial purification of native *Pf*MAG. Purification of parasite crude extract using HiTrap Capto Q column where unbound fractions were collected (**a**). Active fractions from HiTrap Capto Q column were purified using HiTrap Capto S column (**b**). Pooled active fractions from HiTrap Capto S column were purified using HiTrap Heparin HP column (**c**)

Native *Pf*MAG activity was monitored according to *P. falciparum* developmental stages. Relative to hAAG, native *Pf*MAG activity was 3.1, 10.8 and 12.3% in ring, trophozoite and schizont stage, respectively. Crude extract of *P. falciparum* trophozoite and schizont stages from synchronized culture were subjected to purification of *Pf*MAG employing sequential anion exchange, cation exchange and heparin affinity chromatography. The results of the partial purification of PfMAG are summarized in Table 2 and its purification profile was demonstrated in Fig. 1. Ultimately a partially purified enzyme was eluted Hitrap Heparin affinity column employing a linear 0.3–0.6 M KCl gradient (Fig. 1). Two milliliter of parasite pellet yielded 140 μg of 38-fold purified *Pf*MAG, specific activity of 147 U/mg protein. These results indicated existence of a functionally active *Pf*MAG that increased with parasite development.

**Table 2:** Partial purification of native MAG from crude extract of *Plasmodium falciparum* K1 strain

Fraction	Total activity (U)	Total protein (mg)	Specific activity (U/mg)	Yield (%)	Purification fold
Crude extract	41.6	10.6	3.9	100	–
HiTrap Capto Q (unbound)	25.4	3.7	6.8	61.0	1.7
HiTrap Capto S	26.0	0.3	96.2	62.4	24.6
HiTrap Heparin	20.6	0.1	147.4	49.6	37.7

### Expression of *PfMAG* during asexual stage development

**Fig. 2:**
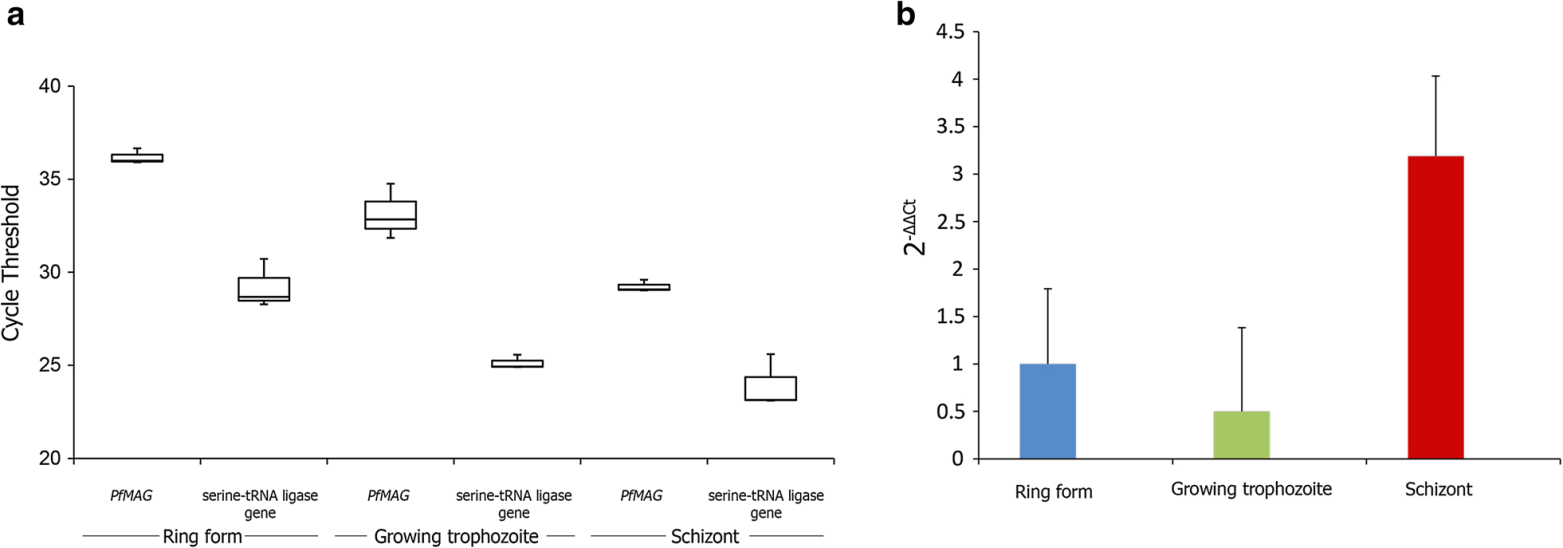
Expression of *PfMAG* during asexual stage development. **a** PCR cycle threshold (C_t_) values of expression levels of *PfMAG* and *serine*-*tRNA ligase* gene are shown as mean (horizontal line), 25th to 75th percentile (box) and range (vertical) for ring, trophozoite and schizont samples. **b** Fold change in *PfMAG* expression of ring, trophozoite and schizont are shown with their standard deviations

SYBR Green qPCR indicated *PfMAG* mean expression level of trophozoite and schizont stage was 0.5 and 3.2-fold(s) of ring form, respectively (Fig. 2), in keeping with relative enzyme activity being highest in schizonts. It was of interest to note the lack of significant difference in *PfMAG* expression level between ring and trophozoite.

### Analysis of *PfMAG* nucleotide sequence

**Fig. 3:**
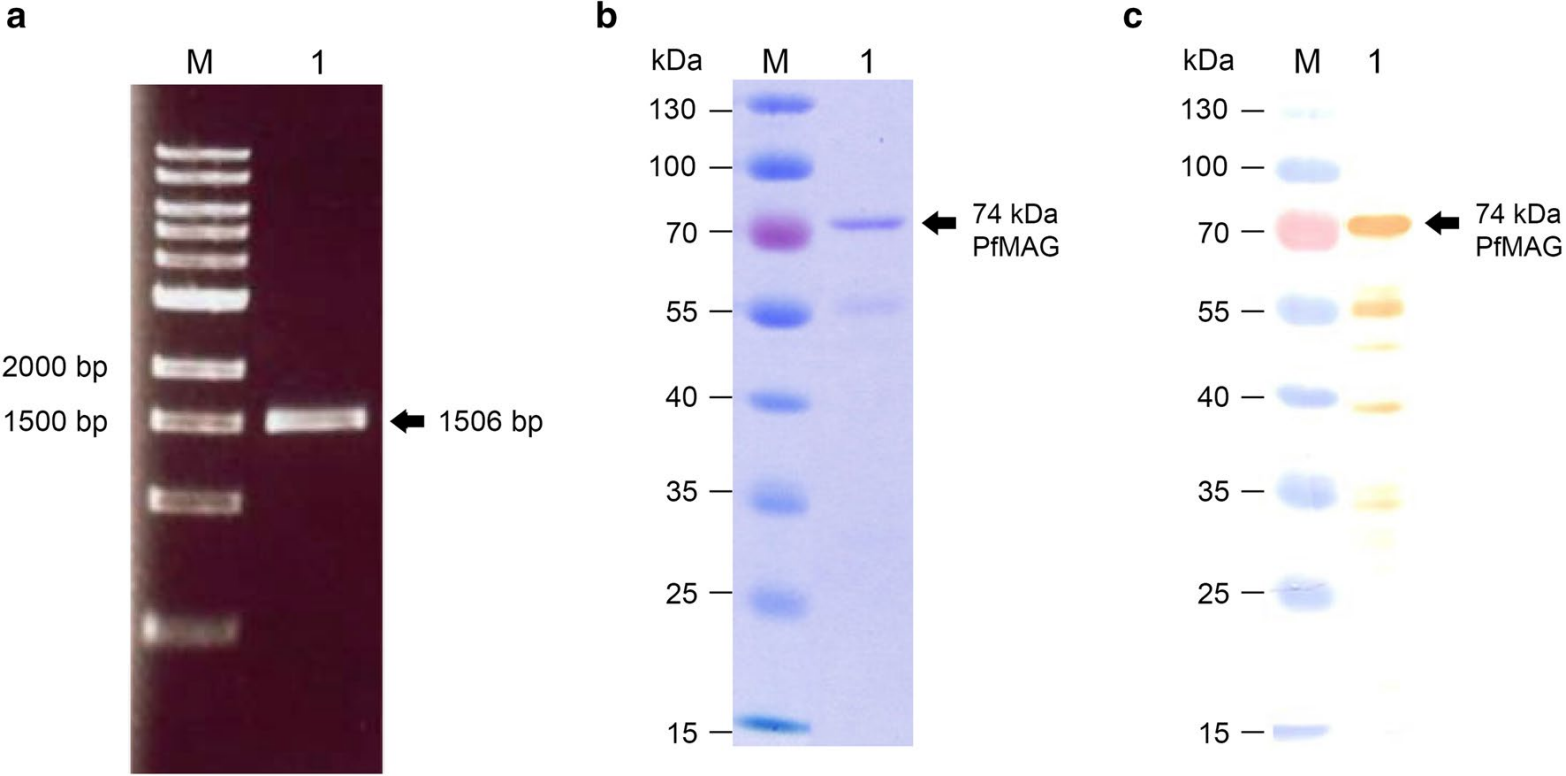
Amplification of *PfMAG* and expression of recombinant *Pf*MAG. **a** PCR amplification *of P. falciparum DNA*-*3*-*methyladenine glycosylase* gene. Lane M, DNA size ladder; lane 1, *PfMAG* amplicon. **b** SDS-PAGE and **c** western blotting of recombinant *Pf*MAG heterologously expressed in *E. coli* LMG 194 at 15 °C for 16 h. Recombinant protein was purified using HisTrap HP column and eluted with a 10–500 mM imidazole linear gradient. Lane M, standard protein size markers; lane 1, purified *Pf*MAG eluted at 500 mM imidazole

The 1506-bp amplicon of K1 *PfMAG* (Fig. 3a) showed 99% identity with that of *P. falciparum* chloroquine-sensitive strain 3D7 strain (NCBI reference sequence XP_001348813.1) due to the presence of 3-nucleotide (AAT) sequence between nt 25 and 29 (Additional file 1: Figure S1), corresponding to an insertion of asparagine (N) at position 9 of the 502-amino acid recombinant enzyme (Additional file 1: Figure S2) but the insertion is not at the active site (Additional file 1: Figure S3), which is conserved among many organisms, including *Plasmodium* spp. The predicted molecular mass of *Pf*MAG is 59.3 kDa with a pI of 9.07. There is only 16% amino acid sequence similarity of *Pf*MAG compared to that of human enzyme. In addition, the predicted amino acid sequence of K1 *Pf*MAG was much different from those of MAGs in other organisms including *Plasmodium* spp. (~ 40%) (Table 3).

**Table 3:** Amino acid sequence similarity of MAG from *Plasmodium falciparum* K1 strain compared to MAGs from other organisms

Organism	Similarity (%)	NCBI protein reference sequence accession number
*P. falciparum 3D7*	99	XP_001348813.1
*P. berghei*	45	XP_679046.1
*P. chabaudi*	44	XP_740495.2
*P. knowlesi*	41	XP_002260194.1
*P. vivax*	40	EDL45990.1
*Mus musculus*	18	NP_034952.2
*Arabidopsis thaliana*	17	NP_187811.1
*Homo sapiens*	16	XP_024306050.1
*Helicobacter pylori*	15	EMJ39070.1
*Escherichia coli*	7	WP_020233157.1
*Saccharomyces cerevisiae*	6	P22134.1

### Simulated 3D structure of PfMAG

**Fig. 4:**
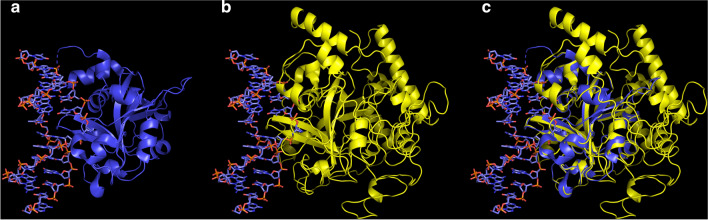
Structural comparison of PfMAG with hAAG. **a** Binding of hAAG (219 residues) with double-strand DNA containing pyrrolidine (PDB 1f6O). Tyr162 intercalates into DNA duplex, flipping pyrrolidine into the active site. **b** Simulated 3D structure of *Pf*MAG (502 residues) using I-TASSER server. Tyr171 intercalates into DNA strand flipping pyrrolidine into the active site. **c***Pf*MAG structure is superimposed on that of hAAG showing similarity of catalytic domain of both proteins and the extended C-terminus of the parasite protein

In view of a lack of an X-ray crystal structure of *Pf*MAG, a simulated 3D structure was constructed based on alignment with that of hAAG (PDB 1F6O) at 41% coverage with RMSD of 2.13 Å (Fig. 4). *Pf*MAG (putatively) contained 20 α-helixes, 11 β-sheets and 31 loops compared to its human orthologue with 14 α-helixes, 8 β-sheets and 22 loops. As expected, the additional N residue is located in a coil region not involved in substrate binding.

### Characterization of recombinant *Pf*MAG

**Fig. 5:**
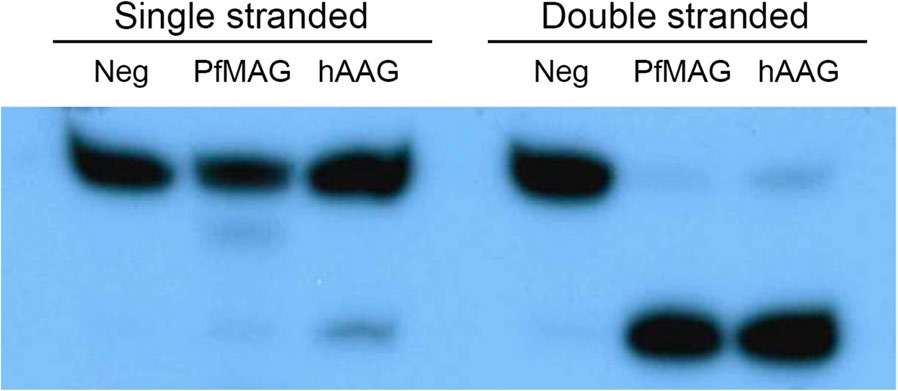
Substrate preference of *Pf*MAG compared to human hAAG in presence of 0.5 μM oligo duplex substrate and 1.5 μM enzyme in the assay reaction. *Pf*MAG cleaves εA in both double-stranded and single-stranded DNA

The affinity-purified recombinant *Pf*MAG had a molecular mass of 74 kDa (Fig. 3b). Western blotting revealed several faint immunoreactive bands of truncated/degraded proteins (~ 55-kDa) accounting for about 15% of total purified protein (Fig. 3c). Specific activity of the affinity-purified recombinant *Pf*MAG was 1309 U/mg. Recombinant *Pf*MAG acted on double-stranded DNA substrate similar to hAAG control (Fig. 5) and converted a 27-mer double-stranded DNA substrate containing εA into a 13-mer product (90% yield) after for 30 min. *Pf*MAG activity on single-stranded substrate was not as clearly demonstrated as with hAAG.

**Fig. 6:**
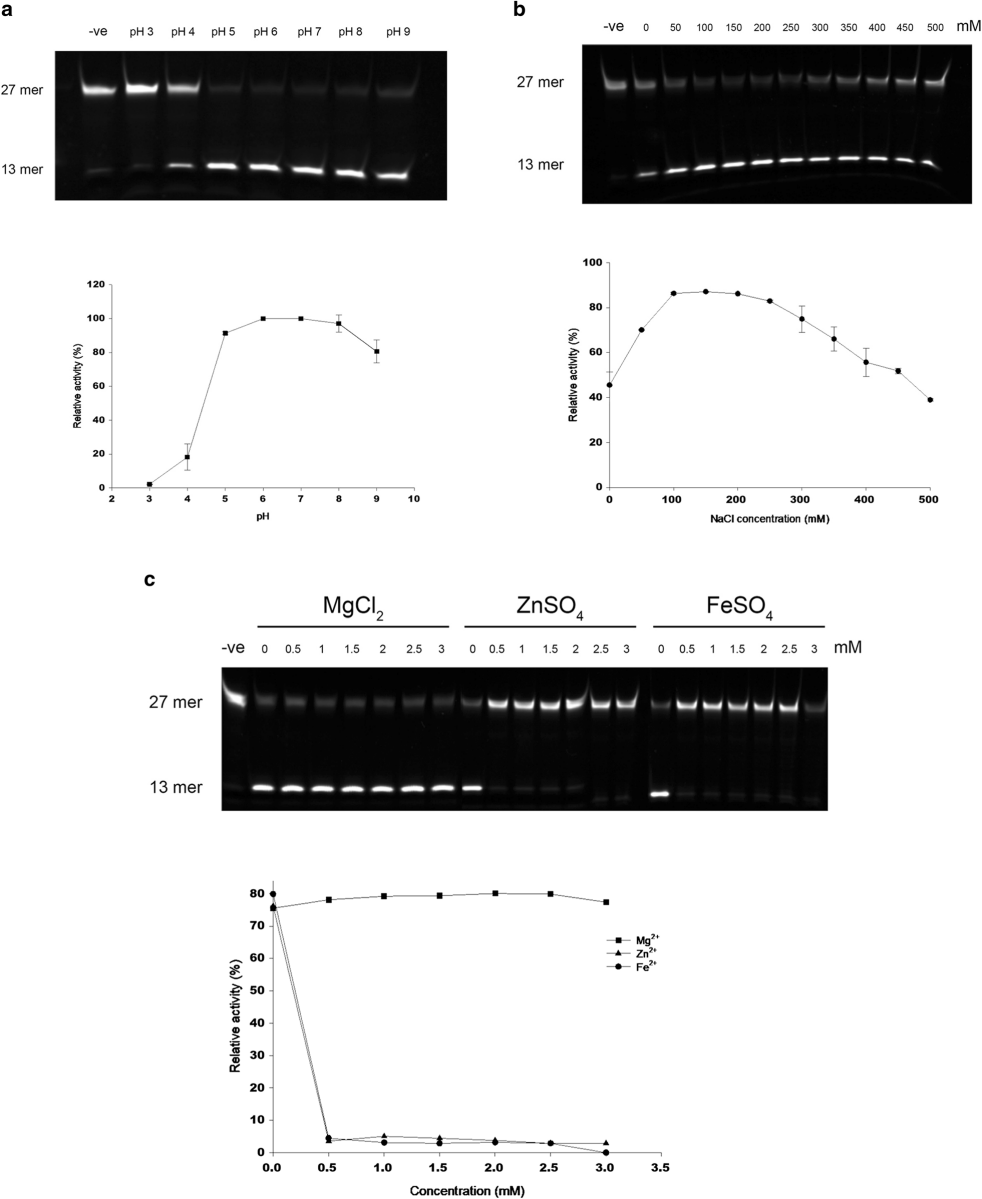
Effects of different buffer conditions on glycosylase activity of recombinant *Pf*MAG. **a** Assay reaction was carried out under standard conditions except buffer used was 50 mM citrate-sodium citrate pH 3-5, phosphate-citrate pH 6-7 and Tris–Cl pH 7–9. **b** Effects of NaCl on glycosylase activity of recombinant *Pf*MAG. Assay reaction was carried out under standard conditions but with 0–500 mM NaCl. **c** Effects of divalent cation on *Pf*MAG activity. The 27-mer double-stranded DNA of εA–T was used as a substrate, and negative control contained no enzyme (− ve)

Recombinant *Pf*MAG had activity over a wide pH range (4–9) (Fig. 6a) but activity was significantly reduced at pH 3, with optimal activity between pH 6 and 7 in a phosphate-citrate buffer. The enzyme had 45.5% relative activity at low-salt concentrations (0–50 mM) and 38.9% at 500 mM compared to optimal concentration of 100–200 mM NaCl that generated 86.3% of product (Fig. 6b). There was no requirement of any divalent cations (Fe^2+^, Mg^2+^ or Zn^2^) for *Pf*MAG activity (Fig. 6c); MgCl_2_ did not affect glycosylase activity up to 3 mM, but iron and zinc sulfate inhibited enzyme activity at 500 μM.

## Discussion

Cellular DNA is constantly damaged by a variety of endogenous metabolites [35]. MAG, a DNA repair enzyme, has multiple substrate specificities, such as methylpurines, ethenopurines and hypoxanthine [36–38]. The enzyme can initiate both short- and long-patch base excision in an alkylated base repair process [39] by intercalating a tyrosine residue between two bases in the DNA strand with subsequent hydrolysis of the *N*-glycosylic bond [40]. Owing to the 80% A–T content of *P. falciparum* whole genome, high numbers of unrepaired alkylated adenine bases constitute a threat to parasite growth and development.

Highest *Pf* MAG activity was found in schizont stages, which correlated with gene expression, but this association was not observed between ring and trophozoite stages. There are reports indicating a large proportion of parasite transcriptional activity, measured during intra-erythrocytic development cycle, does not correlate with protein abundance [41, 42], as observed in mammalian cells where, often time, initiation of translation and not transcript abundance is the main determinant of protein levels [43]. In *Arabidopsis thaliana*, expression of DNA-3-methyladenine glycosylase is also rapidly elevated in dividing tissues and correlates with DNA replication [44]. The human *N*-methylpurine DNA glycosylase (MPG) orthologue is overexpressed in several types of cancers [45].

Low yield from purification of native *Pf*MAG precluded any further characterization of the parasite enzyme other than determination of molecular mass and purity. As in many other studies of malaria parasite enzymes, heterologous expression and affinity purification of recombinant proteins is the recourse in lieu of labour-intensive, large-scale parasite cultivation. The presence of small protein fragments from *Escherichia coli*-expression suggests use of a eukaryote expression host might improve yield and quality of the recombinant protein.

Surprisingly, K1 *Pf*MAG contains an extra asparagine residue at codon 9 compared to 3D7 *Pf*MAG, but based on sequence location and that from a simulated 3D structure, this indel mutation does not appear to affect enzyme activity. Interestingly, *Pf*MAG is nearly twofold larger than of hAAG [46]. The simulated 3D structure of the parasite enzyme shows the extended region consisting of 8 α-helices, 3 β-sheets and 11 loops located at the C-terminus of the parasite protein, and this additional sequence does not bear homology with any other orthologues.

Unlike hAAG, *Pf*MAG was less capable of acting on single-stranded DNA substrate. HAAG is able to excise εA from single-stranded DNA albeit at low efficiency [38], suggesting the possible role of other parasite glycosylase(s) in the repair of these frequent lesions in single-stranded DNA transiently generated during replication and transcription. For instance, in *Escherichia coli*, 3-methyladenine glycosylase has been shown to remove 3-methyladenine from single-stranded DNA [47], and bovine uracil DNA glycosylase [48] and human single-strand selective monofunctional uracil DNA glycosylase [49] also excises uracil from single-stranded DNA substrate.

Recombinant *Pf*MAG functioned over a wide range of pH compared to human and *Saccharomyces pombe* orthologues that show an extremely narrow range of optimal pH (7.5–7.6) [50, 51]. However, *Pf*MAG demonstrated optimal activity in salt concentrations comparable to other MAG orthologues, e.g., 100 mM NaCl and KCl for human *and S. pombe* enzyme, respectively [50, 51], but higher concentrations (250–500 mM) inhibited activity in a dose dependent manner for all three enzymes. The roles of high-salt concentration in inhibiting glycolytic activity are variously attributed to high ionic strength, conformational changes affecting stability and/or solubility and binding of anions to catalytic site [52, 53].

Similar to other DNA glycosylases, *Pf*MAG did not require Mg^2+^ or any other cofactor for damage recognition and/or excision in the assay reaction [54]. *Pf*MAG was not affected by MgCl_2_ even up to 3 mM, which was different from a previous study where MgCl_2_ is able to stimulate and inhibit enzyme activity in a biphasic manner, the latter effect attributed to interference with substrate binding [55]. On the other hand, Fe^2+^ and Zn^2+^ were inhibitory at micromolar concentrations (Fig. 6c), with previous observation that human *N*-methylpurine-DNA glycosylase contains an amino acid residue at the active site with a potential to bind Zn^2+^, thereby interfering with the catalytic process [56].

## Conclusion

Highest levels of *Pf*MAG activity and its gene expression were demonstrated in schizont compared to ring and trophozoite stages. Recombinant *Pf*MAG preferentially acted on double- rather than single-strand DNA, and had a molecular mass twice that of the human enzyme, a broad pH range of activity, optimal activity at 100 mM NaCl, but higher concentrations were inhibitory, and no requirement for Mg^2+^ cofactor but Fe^2+^ and Zn^2+^ were inhibitory in micromolar range. Exploiting characteristics different from those of the human enzyme should provide insights into identifying compounds specifically targeting *Pf*MAG, which could be developed into a potential novel anti-malarial.

## Supplementary information

**Additional file 1: Figure S1.** Comparison of nucleotide sequence of *MAG* of *Plasmodium falciparum* K1 with 3D7 strain. **Figure S2.** Comparison of deduced amino acid sequence of MAG of *Plasmodium falciparum* K1 with 3D7 strain. **Figure S3.** Amino acid sequence alignment of DNA-3-methyladenine glycosylase and active site region.

## Data Availability

Data supporting results in the article are available from the corresponding author upon request.

## Abbreviations

6-FAM: 6-Carboxyfluorescein
Asn: Asparagine
Glu: Glutamic acid
His: Histidine
kDa: Kilodalton
Met: Methionine
Try: Tyrosine
WHO: World Health Organization
