# Supplementary material for: Molecular characterization of Plasmodium falciparum DNA-3-methyladenine glycosylase
Source: Malar J. 2020 Aug 6;19:284. doi: 10.1186/s12936-020-03355-w (PMC7409487; doi:10.1186/s12936-020-03355-w)
Supplement: Supplementary file 1 — Additional file 1: Figure S1. Comparison of nucleotide sequence of MAG of Plasmodium falciparum K1 with 3D7 strain. Figure S2. Comparison of deduced amino acid sequence of MAG of Plasmodium falciparum K1 with 3D7 strain. Figure S3. Amino acid sequence alignment of DNA-3-methyladenine glycosylase and active site region. [file 12936_2020_3355_MOESM1_ESM.docx]

**Figure S1.** A comparison of nucleotide sequencing of *PfMAG* of *P. falciparum* K1 to strain

3D7.

**>ref|XM_001348777.1| Plasmodium falciparum 3D7 DNA-3-methyladenine glycosylase, putative (PF14_0639) mRNA, complete cds**

Length=1506

Score = 2761 bits (1495), Expect = 0.0

Identities = 1503/1506 (99%), Gaps = 3/1506 (0%)

Strand=Plus/Plus

Query 1 ATGGAAAAAATGAACGAATTCTCGaataataataataataataataataatGATGATGAT 60

|||||||||||||||||||||||| |||||||||||||||||||||||||||||||||

Sbjct 1 ATGGAAAAAATGAACGAATTCTCG---AATAATAATAATAATAATAATAATGATGATGAT 57

Query 61 GATAGAGGTTCACTAAAATTAAAACAATTACGTAATTCTATTAAGGGAAAGAAAAGAAAA 120

||||||||||||||||||||||||||||||||||||||||||||||||||||||||||||

Sbjct 58 GATAGAGGTTCACTAAAATTAAAACAATTACGTAATTCTATTAAGGGAAAGAAAAGAAAA 117

Query 121 CATGATAGTATAACAAAAGACAATGATATAATAAATGCATCCAAGGTTATTATaaaaaaa 180

||||||||||||||||||||||||||||||||||||||||||||||||||||||||||||

Sbjct 118 CATGATAGTATAACAAAAGACAATGATATAATAAATGCATCCAAGGTTATTATAAAAAAA 177

Query 181 aaTGTTATGACGACACAACTGTTTGATGATATTAATAAATTGAGTTATGTATATTTCTTA 240

||||||||||||||||||||||||||||||||||||||||||||||||||||||||||||

Sbjct 178 AATGTTATGACGACACAACTGTTTGATGATATTAATAAATTGAGTTATGTATATTTCTTA 237

Query 241 TTAAAATATTATTTTGATAATAATCCATTGAAAATATTAAATGAAAATTTCTATCTTAAA 300

||||||||||||||||||||||||||||||||||||||||||||||||||||||||||||

Sbjct 238 TTAAAATATTATTTTGATAATAATCCATTGAAAATATTAAATGAAAATTTCTATCTTAAA 297

Query 301 GATGATGTATTATATATTACAGAAATATTAATAGGTCATATTTTATGGGTTTTTGACGAG 360

||||||||||||||||||||||||||||||||||||||||||||||||||||||||||||

Sbjct 298 GATGATGTATTATATATTACAGAAATATTAATAGGTCATATTTTATGGGTTTTTGACGAG 357

Query 361 AAACAAAATAAATTATATGGTTCTAGGATTATTGAATTGGAATCTTATAACGGTGTAAAT 420

||||||||||||||||||||||||||||||||||||||||||||||||||||||||||||

Sbjct 358 AAACAAAATAAATTATATGGTTCTAGGATTATTGAATTGGAATCTTATAACGGTGTAAAT 417

Query 421 GATAAAGCATCACATGCTTATAATAATAGAAAAACAAATAGGAATATGTCTATGTTTGAA 480

||||||||||||||||||||||||||||||||||||||||||||||||||||||||||||

Sbjct 418 GATAAAGCATCACATGCTTATAATAATAGAAAAACAAATAGGAATATGTCTATGTTTGAA 477

Query 481 AAAGGTGGAATCAGTTATGTATATTTATGTTATGGTATTCATAATTGTTTAAATATTGTT 540

||||||||||||||||||||||||||||||||||||||||||||||||||||||||||||

Sbjct 478 AAAGGTGGAATCAGTTATGTATATTTATGTTATGGTATTCATAATTGTTTAAATATTGTT 537

Query 541 ACAAATATTCAAAATATACCTGATGCAATTTTAGTTAGATCAACTGAACCATTGTATAAT 600

||||||||||||||||||||||||||||||||||||||||||||||||||||||||||||

Sbjct 538 ACAAATATTCAAAATATACCTGATGCAATTTTAGTTAGATCAACTGAACCATTGTATAAT 597

Query 601 ATTGAATATTTTTTATCAAATAAATTTGAAAAAATATCTCAATATCTTTTGTCAAATTCT 660

||||||||||||||||||||||||||||||||||||||||||||||||||||||||||||

Sbjct 598 ATTGAATATTTTTTATCAAATAAATTTGAAAAAATATCTCAATATCTTTTGTCAAATTCT 657

Query 661 ATATTCATACataaaaatattaaagaacaacaaaaaaataattcaacaccaaaaaataaa 720

||||||||||||||||||||||||||||||||||||||||||||||||||||||||||||

Sbjct 658 ATATTCATACATAAAAATATTAAAGAACAACAAAAAAATAATTCAACACCAAAAAATAAA 717

Query 721 aGGAACCAACAAGGGAACATATCAAATAAAGATGGAAATATTATTAATGAAACAAATTAC 780

||||||||||||||||||||||||||||||||||||||||||||||||||||||||||||

Sbjct 718 AGGAACCAACAAGGGAACATATCAAATAAAGATGGAAATATTATTAATGAAACAAATTAC 777

Query 781 CTAACAAAAATTGATGAATTAATTAATATATTTAAAATGATAAAGAAAAAGCAATTGTTG 840

||||||||||||||||||||||||||||||||||||||||||||||||||||||||||||

Sbjct 778 CTAACAAAAATTGATGAATTAATTAATATATTTAAAATGATAAAGAAAAAGCAATTGTTG 837

Query 841 AAATTATGTAGTGGTCCTGGATGTGTAACAAAAAGTCAAGATATAACCAGGCAAGATGAT 900

||||||||||||||||||||||||||||||||||||||||||||||||||||||||||||

Sbjct 838 AAATTATGTAGTGGTCCTGGATGTGTAACAAAAAGTCAAGATATAACCAGGCAAGATGAT 897

Query 901 AAACAATAtttttttGTGGGCACGAATATTGAGAAGGaaaacaaaacaaatacaattaat 960

||||||||||||||||||||||||||||||||||||||||||||||||||||||||||||

Sbjct 898 AAACAATATTTTTTTGTGGGCACGAATATTGAGAAGGAAAACAAAACAAATACAATTAAT 957

Query 961 aataataataataataaaagaaataattgttcgaaccttcagaaaaattcaaaatatttc 1020

||||||||||||||||||||||||||||||||||||||||||||||||||||||||||||

Sbjct 958 AATAATAATAATAATAAAAGAAATAATTGTTCGAACCTTCAGAAAAATTCAAAATATTTC 1017

Query 1021 aataataatGATTATATaaaaaaaaaCAATAACAAAAGTAACAATATACATAGCGATAAT 1080

||||||||||||||||||||||||||||||||||||||||||||||||||||||||||||

Sbjct 1018 AATAATAATGATTATATAAAAAAAAACAATAACAAAAGTAACAATATACATAGCGATAAT 1077

Query 1081 GATTGTTCTATAGATATAAGTAATTGCACAAAAGAAAACAAACAAACAAATCATTGCAAG 1140

||||||||||||||||||||||||||||||||||||||||||||||||||||||||||||

Sbjct 1078 GATTGTTCTATAGATATAAGTAATTGCACAAAAGAAAACAAACAAACAAATCATTGCAAG 1137

Query 1141 AACGAATATATTCAAGATCATGTGAACTATAATGAACTAATGGAATACTATTATTTTTTA 1200

||||||||||||||||||||||||||||||||||||||||||||||||||||||||||||

Sbjct 1138 AACGAATATATTCAAGATCATGTGAACTATAATGAACTAATGGAATACTATTATTTTTTA 1197

Query 1201 GGTAACATTCATGAAATACACAAAAGCAGGTTTTTTATTACTCTTTGTCCATCCATAAAT 1260

||||||||||||||||||||||||||||||||||||||||||||||||||||||||||||

Sbjct 1198 GGTAACATTCATGAAATACACAAAAGCAGGTTTTTTATTACTCTTTGTCCATCCATAAAT 1257

Query 1261 GATATAATGCttttttttGAAAACCTAAATAATCCTACATACCAAAACAAACATAATTAT 1320

||||||||||||||||||||||||||||||||||||||||||||||||||||||||||||

Sbjct 1258 GATATAATGCTTTTTTTTGAAAACCTAAATAATCCTACATACCAAAACAAACATAATTAT 1317

Query 1321 ATATACAATATATTTAATGAACATAAAAATGTATTATTACAATATTTCCATTTTATGAAA 1380

||||||||||||||||||||||||||||||||||||||||||||||||||||||||||||

Sbjct 1318 ATATACAATATATTTAATGAACATAAAAATGTATTATTACAATATTTCCATTTTATGAAA 1377

Query 1381 TGGGATAAAGAAAATTTTATTGTTCAAAAGGATAAAAGGATAGGAATAAGTTATGCCCAA 1440

||||||||||||||||||||||||||||||||||||||||||||||||||||||||||||

Sbjct 1378 TGGGATAAAGAAAATTTTATTGTTCAAAAGGATAAAAGGATAGGAATAAGTTATGCCCAA 1437

Query 1441 GAAGCTGCCCTATATGATTATCGATTTTTATTAAAGAATCATCCATCCGTATCTATTTTT 1500

||||||||||||||||||||||||||||||||||||||||||||||||||||||||||||

Sbjct 1438 GAAGCTGCCCTATATGATTATCGATTTTTATTAAAGAATCATCCATCCGTATCTATTTTT 1497

Query 1501 CCCAAA 1506

||||||

Sbjct 1498 CCCAAA 1503

**Figure S2.** Amino acid sequence of PfMAG of *P. falciparum* K1 in comparison to strain

3D7.

DNA-3-methyladenine glycosylase, putative [*Plasmodium falciparum* 3D7]

**Sequence ID:** [ref|XP_001348813.1|](http://www.ncbi.nlm.nih.gov/protein/124810257?report=genbank&log$=protalign&blast_rank=5&RID=RP2HJDES01R)**Length:** 501**Number of Matches:** 1

See 3 more title(s)

**Related Information**

[Gene](http://www.ncbi.nlm.nih.gov/gene?term=124810257%5BPUID%5D%20OR%2023497713%5BPUID%5D&RID=RP2HJDES01R&log$=genealign&blast_rank=5)-associated gene details

[Map Viewer](http://www.ncbi.nlm.nih.gov/mapview/map_search.cgi?direct=on&gbgi=124810257&THE_BLAST_RID=RP2HJDES01R&log$=mapalign&blast_rank=5)-aligned genomic context

[Identical Proteins](http://www.ncbi.nlm.nih.gov/protein?LinkName=protein_protein_identical&from_uid=124810257&RID=RP2HJDES01R&log$=identprotalign&blast_rank=5)-Proteins identical to the subject

**Range 1: 1 to 501**[GenPept](http://www.ncbi.nlm.nih.gov/protein/124810257?report=genbank&log$=protalign&blast_rank=5&RID=RP2HJDES01R&from=1&to=501)[Graphics](http://www.ncbi.nlm.nih.gov/protein/124810257?report=graph&rid=RP2HJDES01R%5B124810257%5D&tracks=%5Bkey:sequence_track,name:Sequence,display_name:Sequence,id:STD1,category:Sequence,annots:Sequence,ShowLabel:true%5D%5Bkey:gene_model_track,CDSProductFeats:false%5D%5Bkey:alignment_track,name:other%20alignments,annots:NG%20Alignments%7CRefseq%20Alignments%7CGnomon%20Alignments%7CUnnamed,shown:false%5D&v=0:526&appname=ncbiblast&link_loc=fromHSP)

Next Match

| **Score** | **Expect** | **Method** | **Identities** | **Positives** | **Gaps** |
| --- | --- | --- | --- | --- | --- |
| 994 bits(2570) | 0.0 | Compositional matrix adjust. | 501/502(99%) | 501/502(99%) | 1/502(0%) |
|  |  |  |  |  |  |

Previous Match

Alignment statistics for match #1

Query 1 MEKMNEFSNNNNNNNNNDDDDRGSLKLKQLRNSIKGKKRKHDSITKDNDIINASKVIIKK 60

MEKMNEFSNNNNNNNN DDDDRGSLKLKQLRNSIKGKKRKHDSITKDNDIINASKVIIKK

Sbjct 1 MEKMNEFSNNNNNNNN-DDDDRGSLKLKQLRNSIKGKKRKHDSITKDNDIINASKVIIKK 59

Query 61 NVMTTQLFDDINKLSYVYFLLKYYFDNNPLKILNENFYLKDDVLYITEILIGHILWVFDE 120

NVMTTQLFDDINKLSYVYFLLKYYFDNNPLKILNENFYLKDDVLYITEILIGHILWVFDE

Sbjct 60 NVMTTQLFDDINKLSYVYFLLKYYFDNNPLKILNENFYLKDDVLYITEILIGHILWVFDE 119

Query 121 KQNKLYGSRIIELESYNGVNDKASHAYNNRKTNRNMSMFEKGGISYVYLCYGIHNCLNIV 180

KQNKLYGSRIIELESYNGVNDKASHAYNNRKTNRNMSMFEKGGISYVYLCYGIHNCLNIV

Sbjct 120 KQNKLYGSRIIELESYNGVNDKASHAYNNRKTNRNMSMFEKGGISYVYLCYGIHNCLNIV 179

Query 181 TNIQNIPDAILVRSTEPLYNIEYFLSNKFEKISQYLLSNSIFIHKNIKEQQKNNSTPKNK 240

TNIQNIPDAILVRSTEPLYNIEYFLSNKFEKISQYLLSNSIFIHKNIKEQQKNNSTPKNK

Sbjct 180 TNIQNIPDAILVRSTEPLYNIEYFLSNKFEKISQYLLSNSIFIHKNIKEQQKNNSTPKNK 239

Query 241 RNQQGNISNKDGNIINETNYLTKIDELINIFKMIKKKQLLKLCSGPGCVTKSQDITRQDD 300

RNQQGNISNKDGNIINETNYLTKIDELINIFKMIKKKQLLKLCSGPGCVTKSQDITRQDD

Sbjct 240 RNQQGNISNKDGNIINETNYLTKIDELINIFKMIKKKQLLKLCSGPGCVTKSQDITRQDD 299

Query 301 KQYFFVGTNIEKENKTNTINNNNNNKRNNCSNLQKNSKYFNNNDYIKKNNNKSNNIHSDN 360

KQYFFVGTNIEKENKTNTINNNNNNKRNNCSNLQKNSKYFNNNDYIKKNNNKSNNIHSDN

Sbjct 300 KQYFFVGTNIEKENKTNTINNNNNNKRNNCSNLQKNSKYFNNNDYIKKNNNKSNNIHSDN 359

Query 361 DCSIDISNCTKENKQTNHCKNEYIQDHVNYNELMEYYYFLGNIHEIHKSRFFITLCPSIN 420

DCSIDISNCTKENKQTNHCKNEYIQDHVNYNELMEYYYFLGNIHEIHKSRFFITLCPSIN

Sbjct 360 DCSIDISNCTKENKQTNHCKNEYIQDHVNYNELMEYYYFLGNIHEIHKSRFFITLCPSIN 419

Query 421 DIMLFFENLNNPTYQNKHNYIYNIFNEHKNVLLQYFHFMKWDKENFIVQKDKRIGISYAQ 480

DIMLFFENLNNPTYQNKHNYIYNIFNEHKNVLLQYFHFMKWDKENFIVQKDKRIGISYAQ

Sbjct 420 DIMLFFENLNNPTYQNKHNYIYNIFNEHKNVLLQYFHFMKWDKENFIVQKDKRIGISYAQ 479

Query 481 EAALYDYRFLLKNHPSVSIFPK 502

EAALYDYRFLLKNHPSVSIFPK

Sbjct 480 EAALYDYRFLLKNHPSVSIFPK 501

**Figure S3.** Amino acid sequence alignment of DNA-3-methyladenine glycosylase and the

active site region. Red shading represents active site, green shading represents

DNA binding site, and blue shading represents active sites that bind to DNA

strand.

....|....| ....|....| ....|....| ....|....| ....|....| ....|....| ....|....|

10 20 30 40 50 60 70

**H. sapiens**  .......... .......... .......... .......... .......... .......... .....MVTPA

**M. musculus**  .......... .......... .......... .......... .....MPARG GSARPGRGAL KPVSVTLLPD

**A. thaliana**  .......... .......... .......... .......... .......... .......... ..........

**P. berghei**  .......... .......... .......... .......... .......... .......... .........M

**P. chabaudi**  .......... .......... .......... .......... .......... .......... ..........

**P. falciparum** .......... .......... .......... .......... .......... ........ME KMNEFSNNNN

**P. knowlesi**  .......... .......... .......... .......... .......... .......... .........M

**P. vivax**  MWEGGDIPRC KRCRVRRGRA NGCSPPQGAI CLRFFASVHS RPLPASCSAG EKKRKVVPPT RMNPQSDEIM

**H. pylori**  .......... .......... .......... .......... .......... .......... ..........

**E. coli**  .......... .......... .......... .......... .......... .......... ..........

**S. cerevisiae** .......... .......... .......... .......... .......... .......... ..........

....|....| ....|....| ....|....| ....|....| ....|....| ....|....| ....|....|

80 90 100 110 120 130 140

**H. sapiens**  LQMKKPKQFC RRMGQKKQRP ARAGQPHSSS DAAQAPAEQP HS.....SSD AAQAPCPRER CLGPPTTPGP

**M. musculus**  TEQPPFLGRA RRPGNARAGS LVTGYHEVGQ MPAPLSRKIG QK.....KQR LADSEQQQTP KERLLSTPGL

**A. thaliana**  .......... .......... ........MK TPARRSKRVN QE.......E SETNVTTRVV LRTRKTNCSK

**P. berghei**  GKRKATKFQS SSSNILRSED NEEKKKRNKK ENVNKNNAIK IK.....QAT ISKEKKEKQI DDTSNLSYVY

**P. chabaudi**  .......... .......... .......... .......... .......... .......... ..........

**P. falciparum** NNNNDDDDRG SLKLKQLRNS IKGKKRKHDS ITKDNDIINA SK.....VII KKNVMTTQLF DDINKLSYVY

**P. knowlesi**  QNEKRCRKRR GSLVDSAPPA VRKKDRQRGT LKVERNEGTG RTPPPKIKEH KGEEEKRKKT NQLEYMAYVY

**P. vivax**  QNEQRRRKRR GSLAATASSA VTKRDKPGGE EKGKKKKKIK KH.....TIA GANASAKANA SQLESMAYVY

**H. pylori**  .......... .......... .......... .......... .......... .......... ..........

**E. coli**  ......MERC GWVSQDPLYI AYHDNEWGVP ETDSKKLFEM ICLEGQQAGL SWITVLKKRE NYRASFHQFD

**S. cerevisiae** ......MKLK REYDELIKAD AVKEIAKELG SRPLEVALPE KYIARHEEKF NMACEHILEK DPSLFPILKN

....|....| ....|....| ....|....| ....|....| ....|....| ....|....| ....|....|

150 160 170 180 190 200 210

**H. sapiens**  YRSIYFSSPK GHLTRLGLEF FDQPAVPLAR AFLGQVLVRR LPNGTELRG. RIVETEAYLG PEDEAAHSRG

**M. musculus**  RRSIYFSSPE DHSGRLGPEF FDQPAVTLAR AFLGQVLVRR LADGTELRG. RIVETEAYLG PEDEAAHSRG

**A. thaliana**  TRAARVRPDY PLTRTTSESE MKLMPPEFFQ IDALDLAPRL LGKFMRRDNV VLRITEVEAY RPNDSACHGR

**P. berghei**  ILLKYFFENI NIEILNEQFY LQKNVLTITE ILIGHILWVY NPDKNILCGS RIIELESYNG INDKASHAYN

**P. chabaudi**  .......... .......... .......... .......... .......... .......... ..........

**P. falciparum** FLLKYYFDNN PLKILNENFY LKDDVLYITE ILIGHILWVF DEKQNKLYGS RIIELESYNG VNDKASHAYN

**P. knowlesi**  LLMEYFFENN QLTVLNEKFY LQKNVLPITE ALIGQILWVF DKERKKLYGS RITELEAYNG TEDRASHAYN

**P. vivax**  ILMEYFFENN EVTVFTEKFY LQEDVLSVTE ALIGHILWVY DRGKKKLYGS RITELEAYKG SEDKASHAYN

**H. pylori**  .......... .......... .......... .......FKN ANEIDSSLRN SIEWLSNAGE SLKSKMKEYE

**E. coli**  PVKVAAMQEE DVERLVQDAG IIRHRGKIQA IIGNARAYLQ MEKNGEPFAD FVWSFVNHQP QVTQATTLSE

**S. cerevisiae** NEFTLYLKET QVPNTLEDYF IRLASTILSQ QISGQAAESI KARVVSLYGG AFPDYKILFE DFKDPAKCAE

....|....| ....|....| ....|....| ....|....| ....|....| ....|....| ....|....|

220 230 240 250 260 270 280

**H. sapiens**  GRQTPRNRGM FMKPGTLYVY IIYGMYFCMN ISS..QGDGA CVLLRALEPL E......... ..........

**M. musculus**  GRQTPRNRGM FMKPGTLYVY LIYGMYFCLN VSS..QGAGA CVLLRALEPL E......... ..........

**A. thaliana**  FGVTPRTAPV FGPGGHAYVY LCYGLHMMLN IVADKEGVGA AVLIRSCSPV SG........ ..........

**P. berghei**  NKKTNRNIPM FLNGGISYVY LCYGMHNCLN IVTNIENVPD AILIRSIEPI YNIPFFALNK FQDLNEINNL

**P. chabaudi**  .........M FLNGGISYVY LCYGMHNCLN IVTNAENVPD AILIRSIEPI YNIPFFVLNK FQDIKEINDL

**P. falciparum** NRKTNRNMSM FEKGGISYVY LCYGIHNCLN IVTNIQNIPD AILVRSTEPL YNIEYFLSNK FEKISQYLLS

**P. knowlesi**  NKKTNRNATM FGKGGVSYVY LCYGIHNCLN IVTNEENIPD AILVRSLEPF YGTDSILLKR YKIHSGGSML

**P. vivax**  NKKTNRNATM FGRGGVSYVY LCYGIHNCLN IVTNGENIPD AILVRSLEPF YGAHDILLNR YEIHS..G..

**H. pylori**  RFFNDFNTSM RANEQEVKNT LNANTENIKN EVKKLENQMI E......... .......... ..........

**E. coli**  IPTSTHDSDA LSKALKKRGF KFVGTTICYS FMQACGLVND HVVGCCCYPG NKP....... ..........

**S. cerevisiae** IAKCGLSKRK MIYLESLAVY FTEKYKDIEK LFGQKDNDEE VIESLVTNVK GIGPWSAKMF LISGLKRMDV

....|....| ....|....| ....|....| ....|....| ....|....| ....|....| ....|....|

290 300 310 320 330 340 350

**H. sapiens**  .......... .......... ...GLETMRQ LRSTLRKGTA SRVLKDR... .......... .........E

**M. musculus**  .......... .......... ...GLETMRQ LRNSLRKSTV GRSLKDR... .......... .........E

**A. thaliana**  .......... .......... .......MET IQERRGLKTD KPVLLN.... .......... ..........

**P. berghei**  FSSDNFINQK GNNLKNNRKF KIKELDKMNV EKKIDKKNCA NEIIKKSTYL VILQQLETIF KSIKYKQLVK

**P. chabaudi**  FLFDNFIDKK ENNLKNNR.. ...KSQTEEV DNKIDKKNGT NEIIKKNTYL KSLQQLETVF KNIKYKQLVK

**P. falciparum** NSIFIHKNIK EQQKNN.... ...STPKNKR NQQGNISNKD GNIINETNYL TKIDELINIF KMIKKKQLLK

**P. knowlesi**  GR........ .GSSNPSACA VKGKGGRIPI GCTDEHTYDD SNYCMFKENL QRIEKVKEIL KSINIRKIGK

**P. vivax**  GS.......K LGGDSPGRAA CAVEGDAISY DYPDGPTPPQ SNHSLDKEKL QRIDSVKAIL KTINMKKLAK

**H. pylori**  .......... .......... .......... .......... .......... .......... ..........

**E. coli**  .......... .......... .......... .......... .......... .......... ..........

**S. cerevisiae** FAP....... .....EDLGI ARGFSKYLSD KPELEKELMR ERKVVKKSKI KHKKYNWKIY DDDIMEKCSE

....|....| ....|....| ....|....| ....|....| ....|....| ....|....| ....|....|

360 370 380 390 400 410 420

**H. sapiens**  LCSGPSKLCQ ALAINKSFDQ RDLAQDEAVW LERGPLEPSE .......... .......... ..........

**M. musculus**  LCSGPSKLCQ ALAIDKSFDQ RDLAQDDAVW LEHGPLESSS P......... .......... ..........

**A. thaliana**  ...GPGKVGQ ALGLSTEWSH HPLYSPGGLE LLDGGEDVEK VMVG...... .......... ..........

**P. berghei**  LGSGPGRVTK CLGVTRDDDK KEFYFDICND NNISNNQNNK INAKENNQEN LVKAEKVNDS KNWDIKDD..

**P. chabaudi**  LGSGPGRVTK CIGVTRDDDQ KKLYFDISND QDNKINVKEE ...K...... .....INDDN ISNIKKDD..

**P. falciparum** LCSGPGCVTK SQDITRQDDK QYFFVGTNIE KENKTNTINN NNNNKRN..N CSNLQKNSKY FNNNDYIK..

**P. knowlesi**  VCSGPGCVTK CLDITRKDDK ESFFCDFPNY SMEGKNLTLE GKHEGGCAQV GVVTMQSGDA TQVGEQADTK

**P. vivax**  VCSGPGCVTK CLDITRQDDR ASFFSDVPPY PTQEGKLTWE GKPVGGCAAE GGDLPEGAHP ASEGKNVG..

**H. pylori**  .......... .......... .......... .......... .......... .......... ..........

**E. coli**  .......... .......... .......... .......... .......... .......... ..........

**S. cerevisiae** TFSPYRSVFM FILWRLASTN TDAMMKAEEN FVKS...... .......... .......... ..........

....|....| ....|....| ....|....| ....|....| ....|....| ....|....| ....|....|

430 440 450 460 470 480 490

**H. sapiens**  .......... .......PAV VAAARVGVGH AGEWA..... .......... .......... ..........

**M. musculus**  .......... .......AVV VAAARIGIGH AGEWT..... .......... .......... ..........

**A. thaliana**  .......... ........PR VGIDYALPEH VNALW..... .......... .......... ..........

**P. berghei**  .......... ..CSGNFSKI IDPKYYFSYN VNNFLNE... .......... .......... ..........

**P. chabaudi**  .......... ..CSGNFSKK IDQKYYFSYN VNNFLSI... .......... .......... ..........

**P. falciparum** .......... ..KNNNKSNN IHSDNDCSID ISNCTKENKQ TNHCKNEYIQ DHVNYNELME YYYFLGNIHE

**P. knowlesi**  CIAESIELSE GVHPTIEGKN IKPDHCSTCN IRHLQ..... .......... .......... ..........

**P. vivax**  .......... ..HDHCPPCN VRQDHCSPCR LNDLQ..... .......... .......... ..........

**H. pylori**  .......... .......... .......... .......... .......... .......... ..........

**E. coli**  .......... .......... .......... .......... .......... .......... ..........

**S. cerevisiae** .......... .......... .......... .......... .......... .......... ..........

....|....| ....|....| ....|....| ....|....| ....|....| ....|....| ....|....|

500 510 520 530 540 550 560

**H. sapiens**  RKPLRFYVRG SPWVSVVDRV AEQDTQA... .......... .......... .......... ..........

**M. musculus**  QKPLRFYVQG SPWVSVVDRV AEQMDQPQQT ACS.EGLLIV QK........ .......... ..........

**A. thaliana**  ....RFAVAD TPWISAPKNT LKPL...... .......... .......... .......... ..........

**P. berghei**  KKKSRFFISI CPSVEDVLNF YENLNLEKNS DQY.FIIDIY KQYKIYLLKY FEYMKWKKNQ DTIVQRDKRI

**P. chabaudi**  KKKSRFFISI CPTIEDVLNF YENLNLENNS EQD.FIIDIY KQYKIHLLKY FEYMKWKKDG DIIIQRDKRI

**P. falciparum** IHKSRFFITL CPSINDIMLF FENLNNPTYQ NKHNYIYNIF NEHKNVLLQY FHFMKWDKEN .FIVQKDKRI

**P. knowlesi**  ..KSRFFISV CPSTREIINF YEELVSQKRE NQS.YIQQVY GHYKSHLLDY FKCMKWDQEE .MVVQRDKRV

**P. vivax**  ..RSRFFISI CPSPSEVINF YESLVAQKRE KHN.YMQGLY TQHKSHLLEY LDRMKWDQEK .MVVQRDKRV

**H. pylori**  .......... .......... .......... .......... .......... .......... ..........

**E. coli**  .......... .......... .......... .......... .......... .......... ..........

**S. cerevisiae** .......... .......... .......... .......... .......... .......... ..........

....|....| ....|....| ....|...

570 580

**H. sapiens**  .......... .......... ........

**M. musculus**  .......... .......... ........

**A. thaliana**  .......... .......... ........

**P. berghei**  GVAYAEEYAL YDYRFILKNH PSISVLPK

**P. chabaudi**  GVPYAQECAL YEYRFILKNH PSISVLPK

**P. falciparum** GISYAQEAAL YDYRFLLKNH PSVSIFPK

**P. knowlesi**  GVAYAQEAAF YNYRFLLKGH PSISVLPK

**P. vivax**  GVAYAEEAAL YDYRFLLKGH PSISVPPK

**H. pylori**  .......... .......... ........

**E. coli**  .......... .......... ........

**S. cerevisiae** .......... .......... ........
